# *In vitro* evaluation of ruminant feed from West Sumatera based on chemical composition and content of rumen degradable and rumen undegradable proteins

**DOI:** 10.14202/vetworld.2019.1478-1483

**Published:** 2019-09

**Authors:** Ezi Masdia Putri, Mardiati Zain, Lili Warly, Hermon Hermon

**Affiliations:** 1 Department of Animal Nutrition, Faculty of Animal Science Andalas University, Kampus Limau Manis, Padang, West Sumatera, Indonesia; 2 Department of Animal Nutrition, Faculty of Animal Science Andalas University, Kampus Limau Manis, Padang, West Sumatera, Indonesia

**Keywords:** chemical compositions, rumen degradable proteins, rumen undegradable proteins, ruminant, tropical feedstuff

## Abstract

**Aim:**

This research aimed to discover the chemical composition, as well as the content of the degradable and undegradable protein of the ruminant feed commonly used as cattle feed by Indonesian farmers.

**Materials and Methods:**

In this study, *Pennisetum purpureum*, *Leucaena leucocephala*, *Indigofera zollingeriana*, *Gliricidia sepium*, cassava, maize, palm kernel cake, and rice bran were used as feed. Chemical composition was determined by proximate and Van Soest analyses performed in triplicate. Dry matter and organic matter digestibility, as well as the rumen degradable proteins (RDP) and rumen undegradable proteins (RUP) contents, were determined *in vitro* using the Tilley and Terry method.

**Results:**

The results showed that more proteins can be obtained from legumes than from grass or concentrates. The highest protein amount was obtained from *I. zollingerian* (31.22%), while the lowest protein amount was obtained from cassava (3.59%). Dry matter digestibility ranged from 18.53% (rice bran) to 49.21% (*G. sepium*). Organic matter digestibility ranged from 35.71% (cassava) to 59.57% (*I. zollingerian*). Rice bran had the highest RDP from concentrate (73.26%), whereas *I. zollingerian* had the highest RDP from forage (74.72%). The highest RUP from concentrate was obtained from palm kernel cake (61.01%), and the highest RUP from forage was obtained from *L. leucocephala* (49.23%).

**Conclusion:**

The preparation of ruminant livestock rations must be based on RDP and RUP to meet the needs of both the rumen microbes and host animals. Information regarding the RDP and RUP of feeds is still limited, making this study useful in the preparation of ruminant livestock rations based on RDP and RUP ratios.

## Introduction

In the formulation of feed rations, it is very important to pay attention to the nutritional content of each feed. The nutritional value of the feed has a direct effect on the performance and productivity of livestock. The nutritional value of ruminant animal feed is determined by its chemical composition, as well as the rate of its digestion and its digestibility in the rumen of the livestock. The main purpose of feed chemical analysis is to predict the response of livestock to the feed when it is given as rations. Therefore, information about the chemical composition of feed ingredients is essential in the preparation of livestock rations [1].

Studying the degradation of proteins in ruminants is necessary, as consideration must be given to the fact that the proteins are not only used by host animals but also by the microorganisms living in the rumen. Rumen microbes require rumen degradable proteins (RDP) for microbial protein synthesis; on the other hand, host animals utilize rumen undegradable proteins (RUP), which include bypass proteins that pass directly from the post-rumen and microbial proteins synthesized by rumen microbes [2]. RDP is a protein fraction that is degraded in the rumen by rumen microbes for microbial protein synthesis. RDP is rapidly deaminated by the proteolytic enzymes of rumen microbes to produce ammonia and carbon [2,3]. Synchronizing proteins with feed energy maximizes both the use of nutrients by ruminants and microbial protein synthesis [4]. Microbial proteins are used by livestock as a source of amino acids after they are hydrolyzed in the intestine [2,3]. On the other hand, RUP is a protein fraction that is not degraded in the rumen. Instead, it passes to the post-rumen and is digested in the intestine. RUP increases amino acid passage into the duodenum, and its absorption depends on the animal’s ability to digest the RUP in the post-rumen. Although feed can bypass the rumen, feed with low digestibility cannot be utilized by animals [5].

Overall, the feed given to livestock must provide adequate nitrogen for the rumen microbe (RDP), as well as a direct protein source for host animals (RUP). Appropriate RDP and RUP ratios are needed to efficiently optimize livestock production: Feed with higher RUP than RDP result in decreased rumen microbial protein production, lowered production of volatile fatty acids (VFA), and a decrease in the ability of rumen microbes to perform carbohydrate fermentation. Conversely, an excess of RDP results in increased NH_3_ production in the rumen. Energy is then required to convert the excess NH_3_ to urea in the liver. Previously, the National Research Council has released the chemical composition, as well as the RDP and RUP levels of the feedstuff from subtropical areas. However, there is still a need for information regarding the levels of RDP and RUP of the feedstuff commonly used by farmers in tropical areas, since this information is not yet available.

This research aimed to discover the chemical composition, as well as the content of the degradable and undegradable protein of the ruminant feed commonly used as cattle feed by Indonesian farmers.

## Materials and Methods

### Ethical approval

This research did not use any live animals so, ethical approval is not needed.

### Sampling

Plants and food crops often used as ruminant feed components were evaluated in this study. Forage used was Napier grass (*Pennisetum purpureum*), *Leucaena leucocephala*, *Indigofera zollingeriana*, and *Gliricidia sepium*. These were collected from the UPT Faculty of Animal Husbandry, Andalas University. Each sample was dried using sunlight in the greenhouse of the collection site. Drying was continued using an oven set at 60°C for 48 h, and then the sample was cut and mashed using a grinding machine. The concentrates used were cassava (*Manihot esculenta*), maize (*Zea mays*), palm kernel cake (palm oil and *Elaeis guineensis*), and rice bran (paddy and *Oryza sativa*). Rejected cassava, which is waste produced from *Sanjai* (West Sumatera original cassava chips) production and can be used as animal feed, was purchased from Payakumbuh. Rejected cassava is cassava that is not qualified for *Sanjai* production because of the size. Diameter of the Cassava for *Sanjai* is approximately 5-8 cm. The cassava was cut into pieces and dried in the greenhouse of the Faculty of Animal Husbandry, Andalas University. Drying was continued using an oven 60°C for 48 h, and then the sample was cut and mashed using a grinding machine. Corn was obtained from the poultry shop, palm kernel cake was obtained from Payakumbuh, and rice bran was obtained from rice mill hullers.

### Chemical analyses

Each sample was analyzed to determine the contents of dry matter, organic matter, protein, fat, and crude fiber using proximate analysis [6]. Neutral detergent fiber (NDF), acid detergent fiber (ADF), cellulose, hemicellulose, and lignin contents were analyzed using Van Soest analysis [7].

### In vitro method

*In vitro* analysis was carried out to determine the digestibility, the RDP and RUP levels of each feed component, and the rumen characteristics using the Tilley and Terry method [8], performed for 48 h for forage and 24 h for concentrate. Rumen liquor was obtained from slaughterhouse from Pesisir Cattle (a native Indonesian cow raised in West Sumatera) fed with elephant grass and concentrate. Incubation was stopped by immersing the Erlenmeyer flask into ice water to stop the microbial activity, after which pH measurement was carried out using a pH meter. Next, the supernatant was separated from the residue. To do this, the mixture obtained from *in vitro* analysis was put into a centrifuge tube and then centrifuged for 30 min, 3000 rpm, at 4°C. The supernatant was stored in bottles and then frozen until subsequent NH_3_, total VFA, and partial VFA analyses. NH_3_ concentration was determined using the Conway method, total VFA concentration was determined using steam distillation, and partial VFA concentration was determined using gas chromatography. The residue was filtered using a Whatman No. 41 filter paper and then dried in an oven at 60°C. Afterward, it was analyzed for food substance digestibility using the proximate analysis method.

### Statistical analysis

Data obtained from this research were statistically analyzed using SPSS software version 21.0 [9].

## Results

The chemical compositions of the evaluated forages are shown in Table-1. The chemical composition of each feed component was found to be significantly different (p<0.05). The dry matter, organic matter, crude protein, crude fat, crude fiber, NDF, ADF, hemicellulose, cellulose, and lignin contents varied for each feed component, except for ash content which was not found to be significantly different.

**Table 1:** Proximate and Van Soest analysis of forages (% of dry matter).

Parameters	*Pennisetum purpureum*	*Leucaena leucocephala*	*Indigofera zollingeriana*	*Gliricidia sepium*
Dry matter	90.71^c^±0.35	91.81^d^±0.42	89.68^b^±0.23	88.29^a^±0.07
Organic matter	92.48^d^±0.12	89.90^b^±0.21	88.48^a^±0.64	91.30^c^±0.21
Crude protein	13.13^a^±0.53	25.47^c^±0.25	31.22^d^±0.25	23.84^b^±0.29
Crude fat	2.29^a^±0.30	4.15^b^±0.91	3.48^b^±0.23	3.96^b^±0.11
Ash	6.82±0.96	9.28±0.51	10.33±0.64	7.69±0.98
NDF	67.17^d^±0.54	32.89^c^±0.86	23.23^a^±0.25	27.61^b^±0.71
ADF	40.10^c^±0.48	25.27^b^±0.95	20.78^a^±0.07	22.40^ab^±2.04
Hemicellulose	27.07^c^±1.02	7.62^b^±1.82	2.45^a^±0.26	5.21^ab^±1.33
Cellulose	5.7^a^±0.28	11.38^b^±2.72	4.52^a^±1.28	6.46^ab^±0.75
Lignin	4.25^a^±0.21	11.13^b^±3.22	4.32^a^±1.07	7.72^ab^±0.21

^a,b,c,d^Significantly different in a row (p<0.05). The amount of samples of individual feeds that have been analyzed and evaluated was 24 samples. NDF=Neutral detergent fiber, ADF=Acid detergent fiber

The chemical compositions of the concentrates are shown in Table-2. The chemical composition of each feed component was found to be significantly different (p<0.05). The dry matter, organic matter, crude protein, crude fat, ash, and crude fiber contents varied for each feed.

**Table 2:** Proximate analysis of concentrates (% of dry matter).

Parameters	Cassava	Palm kernel cake	Maize	Rice bran
Dry matter	89.95^c^±0.05	91.53^d^±0.08	83.54^a^±0.07	89.34^b^±0.06
Organic matter	96.98^c^±0.05	95.36^b^±0.39	98.45^d^±0.04	88.14^a^±0.19
Crude protein	3.59^a^±0.35	14.84^c^±0.13	10.98^b^±0.13	11.09^b^±0.26
Crude fat	0.65^a^±0.32	8.20^c^±0.28	3.67^b^±0.29	9.01^c^±0.23
Ash	2.71^b^±0.18	4.25^b^±0.43	1.30^b^±0.11	10.60^a^±2.18
Crude fiber	3.38^b^±0.34	21.60^c^±0.97	1.12^a^±0.14	26.80^d^±0.75

a,b,c,d Significantly different in a row (p<0.05). The amount of samples of individual feeds that have been analyzed and evaluated was 24 samples

Dry matter digestibility, organic matter digestibility, RDP, and RUP composition of the forages and concentrates are shown in Tables-3 and 4. These were found to be significantly different among the forages and concentrates (p<0.05).

**Table 3:** Dry matter digestibility, organic matter digestibility, RDP, and RUP of forages (%).

Parameters	*Pennisetum purpureum*	*Leucaena leucocephala*	*Indigofera zollingeriana*	*Gliricidia sepium*
DMD	42.36^b^±0.42	27.03^a^±0.82	47.14^c^±0.26	49.21^d^±1.41
OMD	49.11^b^±1.75	37.85^a^±1.35	59.47^c^±1.85	58.41^c^±0.47
RDP	64.09^b^±0.94	50.77^a^±0.44	74.72^c^±0.39	66.04^b^±1.33
RUP	35.91^b^±0.94	49.23^a^±0.44	25.28^c^±0.39	33.96^b^±1.33

a,b,c,d Significantly different in a row (p<0.05). The amount of samples of individual feeds that have been analyzed and evaluated was 24 samples. DMD=Dry matter digestibility, OMD=Organic matter digestibility, RDP=Rumen degradable protein, RUP=Rumen undegradable protein

**Table 4:** Dry matter digestibility, organic matter digestibility, RDP content, and RUP content of concentrates (%).

Parameters	Cassava	Palm kernel cake	Maize	Rice bran
DMD	43.94^b^±0.22	41.33^b^±0.06	44.37^b^±0.21	18.53^a^±1.81
OMD	48.22^c^±1.03	45.18^b^±0.39	47.22^c^±0.66	35.71^a^±0.90
RDP	63.87^c^±0.50	38.99^a^±2.27	59.69^b^±1.32	73.26^c^±2.01
RUP	36.13^c^±0.50	61.01^a^±2.27	40.31^b^±1.32	26.74^c^±2.01

^a,b,c,d^Significantly different in a row (p<0.05). The amount of samples of individual feeds that have been analyzed and evaluated was 24 samples. DMD=Dry matter digestibility, OMD=Organic matter digestibility, RDP=Rumen degradable protein, RUP=Rumen undegradable protein

The observed characteristics of rumen fluid after *in vitro* analyses are shown in Tables-5 and 6. It can be seen that among ruminant feed and pH concentration were not found to be significantly different.

**Table 5:** pH, NH_3_, total VFA, and partial VFA concentration of forages.

Parameters	*Pennisetum purpureum*	*Leucaena leucocephala*	*Indigofera zollingeriana*	*Gliricidia sepium*
pH	6.65^a^±0.70	6.90^b^±0.70	6.85^a^±0.70	6.85^a^±0.70
NH_3_production (mM)	21.60^ab^±1.71	17.85^a^±2.02	36.23^c^±3.91	24.59^b^±1.14
Total VFA (mM)	140.78^b^±2.51	100.15^a^±6.85	162.17^c^±0.29	139.68^b^±0.45
Partial VFA (mM)				
Acetate (C_2_)	34.38±15.27	28.71±1.78	28.09±8.32	24.81±5.54
Propionate (C_3_)	11.90±0.98	8.41±0.83	10.07±3.99	6.85±1.82
Butyrate (C_4_)	0.85±0.57	0.39±0.06	1.03±0.28	3.05±3.55

^a,b,c,d^Significantly different in a row (p<0.05). The amount of samples of individual feeds that have been analyzed and evaluated was 24 samples. VFA=Volatile fatty acids

**Table 6:** pH, NH_3_, total VFA, and partial VFA concentration of concentrates.

Parameters	Cassava	Palm kernel cake	Maize	Rice bran
pH	6.85^ab^±0.70	6.90^ab^±0.70	6.80^a^±0.00	6.80^b^±0.70
NH_3_ production (mM)	6.17^a^±0.50	8.48^a^±0.73	8.69^ab^±1.90	11.04^b^±0.29
Total VFA (mM)	67.67^a^±5.83	152.50^d^±3.54	133.37^c^±2.31	118.19^b^±2.57
Partial VFA (mM)				
Acetate (C_2_)	30.79±15.38	25.83±5.07	37.38±11.46	26.43±0.38
Propionate (C_3_)	10.49±7.63	9.41±2.89	11.02±1.05	11.59±0.46
Butyrate (C_4_)	0.51±0.28	0.88±0.68	0.64±0.33	0.75±0.27

^a,b,c,d^Significantly different in a row (p<0.05). The amount of samples of individual feeds that have been analyzed and evaluated was 24 samples. VFA=Volatile fatty acids

## Discussion

Dry matter content of forages ranged from 88.29% (*G. sepium*) to 91.81% (*L. leucocephala*). Organic matter content also ranged from 88.48% (*I. zollingeriana*) to 92.48% (*P. purpureum*). Crude fat ranged from 2.29% (*P. purpureum*) to 4.15% (*L. leucocephala*); however, the crude fat content of legumes was not significantly different. The ash contents ranged from 6.82% (*P. purpureum*) to 10.33% (*I. zollingeriana*) but were not observed to be significantly different among forages. The protein content of each forage varied from 13.13% (*P. purpureum*) to 31.22% (*I. zollingerian*). The protein levels of legume plants were found to be higher than those of grasses and concentrates [1,10,11].

The cell wall content of each feed was also found to be significantly different (p<0.05). Each feed component was analyzed using the Van Soest method to determine the quantities of NDF, ADF, hemicellulose, cellulose, and lignin. Napier grass had the highest cell wall composition when compared to other legumes. The results of this study are consistent with the study of Salama and Ali [12], who reported that Napier grass has high NDF levels compared to other legumes. According to Wilson and Hatfield [13], the rate of feed degradation in the rumen depends on the lignification of plant cell walls. The more a plant cell wall is lignified, the more difficult it is for microbes in the rumen to degrade it. Grass cells are known to be more lignified those of legume plants.

The chemical compositions of each feed component were found to be significantly different (p<0.05). The dry matter, organic matter, crude protein, crude fat, ash, and crude fiber varied for each feed. Dry matter content of concentrates ranged from 83.54% (maize) to 91.53% (palm kernel cake). Organic matter content also ranged from 88.14% (rice bran) to 98.45% (maize). Crude fat content ranged from 0.65% (cassava) to 8.207% (palm kernel cake). Ash content ranged from 1.30% (maize) to 10.60% (rice bran). The protein content of each concentrate ranged from 3.59% (cassava) to 14.84% (palm kernel cake).

Protein levels of legume plants were higher than those of grasses and concentrate [3,10,11]. Feed protein is needed for microbial protein synthesis and for fulfilling the protein requirements of the host animals. Feed with high protein content provides more nitrogen for microbial growth. Nearly 80% of the total rumen microbial population requires nitrogen for microbial protein synthesis [14].

Dry matter and organic matter digestibility were also found to be significantly different (p<0.05). Dry matter digestibility of forages ranged from 27.03% (*L. leucocephala*) to 49.21% (*G. sepium*), whereas organic matter digestibility ranged from 37.85% (*L. leucocephala*) to 59.47% (*I. zollingeriana*). Meanwhile, the dry matter digestibility of concentrates ranged from 18.53% (rice bran) to 44.37% (maize), while the organic matter digestibility ranged from 35.71% (rice bran) to 48.22% (cassava).

The variations in the feed components show the contribution of the feed to livestock needs and the synthesis of microbial proteins. This is consistent with the research of Polyorach and Wanapat [15], who reported that higher levels of feed digestibility increase the available carbon structures used for microbial protein synthesis, a process in which these carbon structures combine with ammonia.

Optimum protein sources for ruminants should be able to provide nitrogen for microbial growth; moreover, it should high bypass proteins, and it should have high biological value [2]. These characteristics ensure that the degradable protein (RDP) and the protein by-pass (RUP) needed for ruminant productivity are supplied.

The levels of degradable proteins (RDP) and non-degradable proteins (RUP) are shown in Table-4. Each feed had varying RDP and RUP levels that differed significantly (p<0.05). The RDP content of feed ranged from 38.99% (palm kernel cake) to 74.22% (*I. zollingeriana*). RDP levels of Napier grass, *I. zollingeriana*, *L. leucocephala*, and *G. sepium* were found to be higher than that of grain (corn). This is consistent with Mehmet and James [16], who reported that the degraded protein fractions are higher in forage than in grain. The RDP of *L. leucocephala* was observed to be lower than *G. sepium* and *I. zollingeriana*, which is caused by the tannins contained in each plant. A feed with higher tannin contents has lower digestibility in the rumen, as the tannins can form complex bonds with proteins, which in turn makes microbial degradation in the rumen difficult.

Hydrolyzed tannins and condensed tannins can be tolerated to a certain extent by rumen microbes, but condensed tannins bind more to proteins in the rumen compared to hydrolyzed tannins. These microbes include *Clostridium proteoclasticum*, *Ruminococcus albus*, and *Streptococcus gallolyticus* [17]. Based on the results of laboratory analysis, total tannin levels were 0.21% in *L. leucocephala*, 0.41% in *G. sepium*, and 1.66% in *I. zollingeriana*. Even though *L. leucocephala* had low tannin levels, it was still slightly degraded in the rumen [18,19] because *L. leucocephala* has more condensed tannins (0.075%) than *G. sepium* (0.039%) and *I. zollingeriana* (0.027%). This degraded protein will be utilized in the form of NH_3_ (ammonia). Ammonia released in the rumen is partly utilized by microbes to synthesize microbial proteins. If ammonia is released quickly, ammonia is absorbed through the rumen wall; consequently, very little ammonia can be used by bacteria.

The dynamics of microbial protein synthesis in the rumen depend on factors such as rumen pH values, rumen microbes, dry organic matter of feeds, feeding, fermented energy source feeds, N compounds, synchronization of feed energy and protein sources, forage and concentrate ratios, and the rate of passage in the rumen [20]. Crude protein is an essential component for microbial protein synthesis, as it serves as a source of nitrogen for the rumen microbes when there are sufficient nitrogen concentrations and when the protein is not used as an energy source [21].

The characteristics of rumen fluid analyzed *in vitro* are shown in Tables-4 and 5. Among the ruminant feed, pH concentrations were not significantly different. Noting the rumen condition, especially rumen pH, is important [22] as it plays an important role in the ability of rumen microbes to degrade feed proteins [23]. This study has demonstrated that the pH of the rumen fluid for fermenting feed is still within the normal range of 6.65-6.90. Optimum pH for normal rumen condition is 5.5-6.9, with a dry material content of 10-13% and a temperature of 38-41°C [24].

The results show a variation in rumen NH_3_ production for each feed. NH_3_ production from this study ranged from 6.17 to 36.23 mM. The range of NH_3_ production can support microbial protein synthesis needed for ruminant production; therefore, NH_3_ parameters need to be considered. This is consistent with McDonald *et al*. [25], who reported that microbial protein production increases in the NH_3_ 6-21 mMol production.

The total VFA concentrations in this study ranged from 67.67 to 162.17 mM and were found to be significantly different (p<0.05). The highest total VFA was recorded from *I. zollingeriana*, which was 162.17 mM. The high level of total VFA was influenced by the *I. zollingeriana* protein, which was at 31.22%. This is in accordance Sairullah *et al*. [26], who reported that feed with high protein content significantly increase VFA production because the protein level can support microbial protein synthesis until its peak; as such, microbial feed fermentation also increases.

Partial VFA represents the concentration of each free fatty acid present in the rumen fluid. Based on Table-5, the concentrations of acetate, propionate, and butyrate were not significantly different among the feed components. It also shows that the acetate concentration of each feed was higher than the concentrations of propionate and butyrate (acetate >propionate >butyrate). These results are consistent with Czerkawski [27], who reported that acetate and propionate, which are rumen fermentation products, are the main components of VFA. Butyrate concentrations are the next-highest, followed by small quantities of other types of VFA such as formic acid, isobutyrate, valerate, isovalerate, and isocaproate. The concentration of acetate ranged from 5.07 to 34.38 mM, propionate ranged from 6.85 to 11.90 mM, and butyrate ranged from 0.39 to 3.05 mM. McDonald *et al*. [25] reported that the concentration of VFA in the rumen depends on the food consumed and the type of ruminants.

## Conclusion

This research has shown the variations in the protein contents of each feed component. The protein content of legumes was observed to be higher than those of grass and concentrates. The levels of dry matter and organic ingredients did not significantly vary between feed ingredients. Elephant grass had the highest fiber fraction compared to other forages. Dry matter and organic matter digestibility varied among feed ingredients. Rice bran had the highest RDP from the concentrates, while *I. zollingerian* had the highest RDP from the forages. The highest RUP from the concentrates was obtained from palm kernel cake, while the highest RUP from the forages was obtained from *L. leucocephala*. Information on the nutritional content of feeds is useful in preparing rations for livestock, as the preparation of ruminant livestock rations must be based on RDP and RUP to meet the needs of both rumen microbes and host animals. Information about RDP and RUP of feeds is still limited; therefore, this study is useful in aiding the preparation of ruminant livestock rations based on RDP and RUP values.

## Authors’ Contributions

EMP, MZ, LW, and HH formulated the experimental design and experimental work at the laboratory. EMP drafted the manuscript and did data analysis under the guidance of MZ, LW, and HH. All the authors read and approved the final version of the manuscript.
